# PRMT1 mediates RANKL-induced osteoclastogenesis and contributes to bone loss in ovariectomized mice

**DOI:** 10.1038/s12276-018-0134-x

**Published:** 2018-08-28

**Authors:** Joo-Hee Choi, Ah-Ra Jang, Dong-il Kim, Min-Jung Park, Seul-Ki Lim, Myung-Sun Kim, Jong-Hwan Park

**Affiliations:** 1 Laboratory Animal Medicine, College of Veterinary Medicine, Chonnam National University, Gwangju, 61186 Republic of Korea; 2 Department of Physiology, College of Veterinary Medicine, Chonnam National University, Gwangju, 61186 Republic of Korea; 3 Department of Molecular and Integrative Physiology, University of Michigan Medical School, Ann Arbor, MI 48109 USA; 4 Microbiology and Functionality Research Group, World Institute of Kimchi, Gwangju, 61755 Republic of Korea; 5 Department of Orthopaedic Surgery, Chonnam National University Medical School, Gwangju, 61469 Republic of Korea

## Abstract

Protein arginine methylation is a novel form of posttranslational modification mediated by protein arginine methyltransferase (PRMTs). PRMT1, a major isoform of the PRMT family, is responsible for various biological functions, including cellular differentiation. Although the important function that PRMT1 plays in various tissues is being increasingly recognized, its role in receptor activation of NF-κB ligand (RANKL)-induced osteoclastogenesis or osteoporosis has not yet been described. Here, we show that PRMT1 is essential for RANKL-induced osteoclastogenesis in vitro and for bone loss in vivo. RANKL treatment increased the expression of PRMT1 and its nuclear localization in bone marrow-derived macrophages (BMDMs) in a c-Jun N-terminal kinase (JNK)-dependent manner. Silencing PRMT1 attenuated RANKL-induced osteoclastogenesis by decreasing tartrate-resistant acid phosphatase (TRAP)-positive cells and inhibiting F-actin ring formation and bone resorption, which was confirmed in a separate experiment using haploinsufficient cells from *PRMT1*^*+/-*^ mice. Our results also revealed that PRMT1 regulates the transcription activity of NF-κB by directly interacting with it in RANKL-treated BMDMs. An in vivo study showed that the haploinsufficiency of PRMT1 reduced the enzyme activity of TRAP and increased the bone mineral density in the metaphysis of ovariectomized (OVX) mice. Finally, treatment with estrogen (E2) downregulated the RANKL-induced expression of PRMT1, suggesting that estrogen may exert an inhibitory effect on osteoclastogenesis by suppressing PRMT1 expression. Our results suggest that PRMT1 plays an important role in the progression of osteoporosis and that it might be a good therapeutic target for postmenopausal osteoporosis.

## Osteoporosis: protein trigger for postmenopausal bone loss identified

A protein that helps trigger bone loss in postmenopausal osteoporosis could be a potential therapeutic target. After the menopause, decreases in estrogen hormone levels can lead to bone diseases including osteoporosis. Osteoporosis occurs when the bone remodeling process breaks down, and bone resorption by cells called osteoclasts outweighs bone formation. In a mouse model of postmenopausal osteoporosis, Jong-Hwan Park at Chonnam National University, Gwangju, South Korea and co-workers identified key players in the progression of the disease. The team focused on factors influencing the RANKL protein, a known controller of bone remodeling. They found that RANKL triggers the formation of osteoclasts via interaction with another protein, PRMT1. Suppression of PRMT1 by estrogen appears to inhibit excessive osteoclast formation, suggesting it could be a potential therapeutic target for treating osteoporosis.

## Introduction

Bone is continuously renewed by replacing old bone with new bone through bone remodeling, which is a turnover process consisting of the interaction and balance between bone-resorbing cells (osteoclasts) and bone-forming cells (osteoblasts)^1^. The potential imbalance between osteoclasts and osteoblasts plays a fundamental role in the pathogenesis of osteoporosis.

Estrogen deficiency in postmenopausal women stimulates osteoclast formation, and this result in the development of postmenopausal osteoporosis^2,3^. It is estimated that estrogen deficiency induced osteoporosis affects 40% of women over the age of 50^4^. Among currently available anti-osteoporotic drugs, postmenopausal estrogen replacement therapy has been shown to have the strongest protective effect against osteoporosis in women. Unfortunately, use of hormone supplementation in postmenopausal women has been limited because of possible increased risks of breast and endometrial cancers with long-term use^5^. Therefore, understanding the cellular and molecular mechanisms that govern changes in the activity of cells associated with bone remodeling may identify potential therapeutic targets for osteoporosis and other bone-associated pathology.

Osteoclasts are derived from undifferentiated cells in a monocyte–macrophage lineage. Specifically, they are simultaneously stimulated by two cytokines: macrophage colony-stimulating factor (M-CSF) and receptor activator of nuclear factor-kappa B ligand (RANKL). M-CSF is essential for the survival and proliferation of osteoclast precursors, and RANKL plays a key role in osteoclast differentiation and activation^6^. RANKL-induced activation of RANK on osteoclast progenitor cells leads to the recruitment of tumor necrosis factor (TNF) receptor-associated factor 6 (TRAF6), consequently activating several downstream signaling molecules such as NF-κB and mitogen-activated protein kinases (MAPKs) in early-stage osteoclast differentiation^7^. This signaling cascade leads to the activation of major transcription factors, such as the nuclear factor of activated T cells c1 (NFATc1) and c-fos, a member of the activator protein 1 (AP-1) family of transcription factors^8–10^.

Protein arginine methyltransferases (PRMTs) mediate protein arginine methylation, which is a novel posttranslational modification^11,12^. All PRMTs generate monomethylarginine (MMA); however, they are classified as either type 1 or type 2, depending on the type of dimethylated arginine that they form. Type I PRMTs—including PRMT1, 3 and 4—generate asymmetric dimethylarginine (ADMA) formation^13^. Among these, PRMT1 accounts for >90% of the generation of ADMA^14^. PRMT1 has been described as contributing to the development and progression of cancer, cardiovascular disease and other pathophysiological conditions such as diabetes mellitus and hepatic lipogenesis^15–19^. PRMT1 is also thought to be involved in estrogen deficiency mediated osteoporosis given that an increase in the serum ADMA level is associated with an age-related decrease in the BMD of rats^20^. Moreover, 17β-estradiol decreases the circulating concentration of ADMA in vivo^21^. In addition, serum ADMA levels have been found to be increased in ankylosing spondylitis (AS), a chronic immuno-inflammatory rheumatic disease^22^. Type II PRMTs, including PRMT5 and 7, produce symmetric dimethylarginine (SDMA)^23^. Recently, Dong et al. demonstrated that PRMT5 is an osteoclastogenesis activator and that inhibition of PRMT5 suppresses osteoclast differentiation via downregulation of CXCL10 and RSAD2 in vitro and in vivo^24^. Although the importance of PRMT1 in various tissues has been increasingly recognized, the role of PRMT1 in osteoclastogenesis and bone loss has not yet been described. In the present study, we provide evidence that PRMT1 is critical for RANKL-induced osteoclastogenesis in vitro and that it contributes to bone loss in ovariectomized (OVX) mice, which is a representative animal model for postmenopausal osteoporosis.

## Materials and methods

### Reagents and antibodies

Minimum essential medium alpha medium (α-MEM) and fetal bovine serum (FBS) were purchased from Life Technologies (Gibco BRL, Grand Island, NY, USA). Recombinant mouse M-CSF was purchased from Miltenyi Biotec (Gladbach, Germany). Recombinant mouse sRANKL was obtained from Prospec Biotec (Ness-Ziona, Israel). SB 203580, SP 600125, and PD 98059 were purchased from Enzo Life Sciences (AG, Switzerland). 17β-Estrogen (E2) was obtained from Sigma-Aldrich (St. Louis, MO, USA). Estrogen receptor (ER) antagonist ICI 182,780 was purchased from Tocris Bioscience (Bristol, UK). Antibodies against PRMT1, ASYM24, the regular- or phospho-form of p65, p38, extracellular signal-regulated kinase (ERK) and c-Jun N-terminal kinase (JNK) were purchased from Cell Signaling Technology (Danvers, MA, USA). β-Actin and cathepsin K antibodies were purchased from Santa Cruz Biotechnology (Santa Cruz, CA, USA).

### Osteoclastic differentiation and TRAP staining

To generate osteoclasts, mouse bone marrow cells were isolated from femurs of 6- to 8-week-old mice (Samtako, Gyunggi-Do, Korea). After lysing the red blood cells, the remaining bone marrow cells were incubated at 37 °C for 3 days in 5% CO_2_ in α-MEM supplemented with 10% FBS and 1% penicillin/streptomycin in the presence of M-CSF (30 ng/ml). The bone marrow-derived macrophages (BMDMs) were obtained by removing the floating cells. Adherent cells (BMDMs) were used as osteoclast precursors. The BMDMs were cultured in the presence of M-CSF (30 ng/ml) and RANKL (100 ng/ml) for 3 more days. Osteoclast formation was determined by tartrate-resistant acid phosphatase (TRAP) staining. After 3 days, the cells were fixed with a fixative solution for 30 s, and TRAP staining was performed using a commercial kit (Sigma, MO, USA) according to the manufacturer’s instructions. The number of TRAP-positive multinucleated cells (MNCs, containing more than three nuclei) was counted using a light microscope.

### Animal experiments

PRMT1 haploinsufficiency mice (*PRMT1*^+/-^) on a C57BL/6 background were kindly provided by Dr. Seung-Hoi Koo (Department of Life Science, Korea University, Seoul, Korea). Wild-type (WT) (*PRMT1*^+/+^) mice were mated with heterozygous *PRMT1*^*+/-*^, and the littermates were used in the experiments after genotyping. All mice were housed in a specific pathogen-free condition at a room temperature of 22 ± 1 °C with 50% humidity. Female 7-week-old WT (*n* = 6) and *PRMT1*^*+/-*^ (*n* = 6) mice were OVX by removing the bilateral ovaries through a dorsal approach under general anesthesia with rompun (10 mg/kg) and zoletil (30 mg/kg). Sham surgery was performed in the WT group (*n* = 6) by identifying the bilateral ovaries. After 8 weeks, all mice were sacrificed, and their femora were collected for micro-computed tomography (μCT). For the TRAP assay, bones were fixed in 10% formalin and were decalcified by immersion in 10% ethylenediaminetetra-acetic acid (EDTA) for 10 days. All animal studies were approved by the Institutional Animal Care and Use Committee (IACUC) of Chonnam National University (approval number: CNU IACUC-YB-2016-32).

### Western blotting

Cell pellets were lysed in Mammalian Protein Extraction Reagent (Thermo, IL, USA) containing protease inhibitor cocktail (Sigma, MO, USA) and phosphatase inhibitor cocktail I + II (Sigma, MO, USA). Each fractional protein was extracted according to the manufacturer’s instructions. The protein levels were quantified using the Bradford procedure. Whole-cell extracts (30 μg each) were separated by sodium dodecyl sulfate polyacrylamide gel electrophoresis (SDS-PAGE) and were transferred onto enhanced nitrocellulose membranes. The membranes were then washed with tris buffered saline with tween 20 (TBST), blocked with 5% skim milk for 1 h and incubated with primary antibodies (at dilutions recommended by the supplier) overnight at 4 °C. The membranes were then washed with TBST and incubated with horseradish peroxidase-conjugated secondary antibodies for 2 h at room temperature. Bands were visualized with a Luminescent Image Analyzer (ImageQuant LAS 4000, GE Healthcare, UK) using Amersham ECL^TM^ Western Blotting Detection Reagents (GE Healthcare, UK).

### Bone resorption assays and actin ring staining

For the bone resorption assay, BMDMs were cultured for 4 days with M-CSF (30 ng/ml) in the presence or absence of RANKL (100 ng/ml) on an Osteo assay surface plate (Corning Inc., NY). To quantitate the resorption lacunae, the cells were removed with 20% SDS followed by extensive washing with distilled water and air drying. The areas absorbed on the discs were observed under a microscope (Eclipse Ni-U, Nikon, Japan). To evaluate the actin ring formation by osteoclast-like cells, mature osteoclasts were prepared from BMDMs via treatment with M-CSF (30 ng/ml) in the presence or absence of RANKL (100 ng/ml) for 3 days on a cover glass. The cells were fixed and permeabilized with 0.2% Triton X-100/phosphate-buffered saline (PBS) followed by staining with Alexa Fluor 594-phalloidin (Invitrogen). The cells were then mounted on slides, and the nuclei were stained with 4′, 6-diamidino-2-phenylindole (DAPI) in ProLong Gold Antifade Mounting Medium (Invitrogen, Carlsbad, CA, USA).

### siRNA transfection

Small interfering RNAs (siRNAs) for PRMT1 (Santa Cruz) and control siRNA (Qiagen) were used to silence endogenous PRMT1 expression. Each siRNA (50 nM) was transfected into BMDMs using Lipofectamine^TM^ RNAiMAX reagent (Carlsbad, CA, USA) following the forward transfection method as instructed by the manufacturer.

### Real-time quantitative polymerase chain reaction (qPCR)

Total RNA was extracted from cells using TRIzol, a monophasic solution of phenol and guanidine isothiocyanate purchased from Invitrogen (Carlsbad, CA, USA). Then, 1 μg of RNA was reverse transcribed into complementary DNA (cDNA) using an RT Premix reverse transcription system (AccuPower, Seoul, Korea) with oligo-dT18 primers. Then, qPCR was performed using the cDNA as a template with Power SYBR Green (Applied Biosystems, Warrington, UK). The following primers were used: PRMT1, 5′-CCTCACATACCGCAACTCCA-3′ and 5′-CATCCAGCACCACCTTGTCT-3′; c-fos, 5′-CCAGTCAAGAGCATCAGCAA-3′ and 5′-AAGTAGTGCAGCCCGGAGTA-3′; NFATc1, 5′-CTCGAAAGACAGTGGAGCAT-3′ and 5′-CGGCTGCCTTCCGTCTCATAG-3′; TRAP, 5′-CTGGAGTGCACGATGCCAGCGACA-3′ and 5′-TCCGTGCTCGGCGATGGACCAGA-3′; cathepsin K, 5′-GGCCAACTCAAGAAGAAAAC-3′ and 5′-GTGCTTGCTTCCCTTCTGG-3′; DC STAMP, 5′-CCAAGGAGTCGTCCATGATT-3′ and 5′-GGCTGCTTTGATCGTTTCTC-3′; and β-actin, 5′-AGGCCCAGAGCAAGAGAG-3′ and 5′-TCAACATGATCTGGGTCATC-3′. qPCR data were normalized using β-actin as an endogenous control. Real-time PCR was performed in a Rotor-Gene Q real-time PCR system (Qiagen, Hilden, Germany) using a two-step protocol of 95 °C for 10 s followed by 40 cycles of 60 °C for 60 s.

### Enzyme-linked immunosorbent assay (ELISA)

The concentration of ADMA in the culture supernatant was measured using an ultrasensitive ELISA kit (Eagle Biosciences, Nashua, NH, USA) according to the manufacturer’s instructions.

### Immunofluorescence and confocal microscopy

The cells were washed twice in PBS and fixed for 10 min with 4% paraformaldehyde in PBS. After three washes in PBS, the fixed cells were permeabilized with 0.2% Triton X-100 and 1% bovine serum albumin solution was used for blocking. The cells were incubated with PRMT1 antibody (dilution ratio 1:100) for 15 h at 4 °C. After three washes in PBS, the cells were incubated with anti-rabbit fluorescein isothiocyanate (FITC) secondary antibody (Sigma, MO, USA). Then, the cells were mounted on slides and the nuclei were visualized with DAPI. Immunofluorescence imaging was performed on a Leica TCS SP5 AOBS laser scanning confocal microscope (Leica Microsystems, Heidelberg, Germany) using a Leica 63 × (N.A. 1.4) oil objective located at the Gwangju Center of the Korea Basic Science Institute. Excitation (496 and 405 nm) and emission (500–535, 449–461 nm) were observed for the FITC-conjugated construct and DAPI, respectively. For all experiments, the exposure time was kept the same for all samples.

### Luciferase assay

For the luciferase assay, Raw 264.7 macrophages were transiently co-transfected with pGL4.32-*luc2P*/NF-κB-RE (Promega, WI, USA) and a control or PRMT1 with a β-galactosidase expression vector to normalize the expression of the reporter gene using Lipofectamine 3000 (Invitrogen, Carlsbad, CA, USA). At 48 h post transfection, the culture medium was replaced with medium containing RANKL followed by incubation for 24 h. The cells were lysed with passive lysis buffer. The promoter activities were measured using beetle luciferin and a GLOMAX luminometer. The reagents for measuring luciferase activity were purchased from Promega.

### Immunoprecipitation

RAW 264.7 cells were incubated with or without RANKL (100 ng/ml) in the presence of M-CSF (30 ng/ml) for 24 h. The cells were lysed in non-denaturing lysis buffer composed of 20 mM Tris (pH 7.4), NaCl 150 mM, 1% NP-40, 1 mM EDTA and 5% glycerol. Then, 200 μg of protein was incubated with PRMT1 antibody and 40 μl of protein agarose G under non-denaturing conditions for 24 h at 4 °C. The immunoprecipitates were extensively washed, re-suspended in 2 × sample buffer, boiled for 7 min, and subjected to immunoblotting.

### μCT analysis

A high-resolution Skyscan 1076 system was used to produce the μCT images of the distal femurs. Raw images were reconstructed into serial cross-sectional images with identical thresholds for all samples using Image Reconstruction Software version 3.1. A total of 240 steps of the proximal femur trabecular bones starting from 80 steps away from the epiphyseal plate were manually designated as regions of interest. Femoral morphometric parameters were determined using data analysis software (CTAn). The trabecular morphometry was characterized by measuring bone volume per tissue volume (BV/TV), trabecular number (Tb. N), trabecular thickness (Tb. Th) and bone mineral density (BMD).

### Statistical analysis

All statistical analyses were determined using GraphPad Prism 5 (GraphPad, La Jolla, CA, USA). The statistical values were calculated using Student’s *t*-test to compare the means of two groups. For comparisons between multiple values, one-way analysis of variance (ANOVA) with Tukey’s post-hoc test was used to assess differences between specific groups. A result was considered statistically significant if the *P*-value was <0.05.

## Results

### Expression of PRMT1 is upregulated by RANKL during osteoclast differentiation

**Fig. 1:**
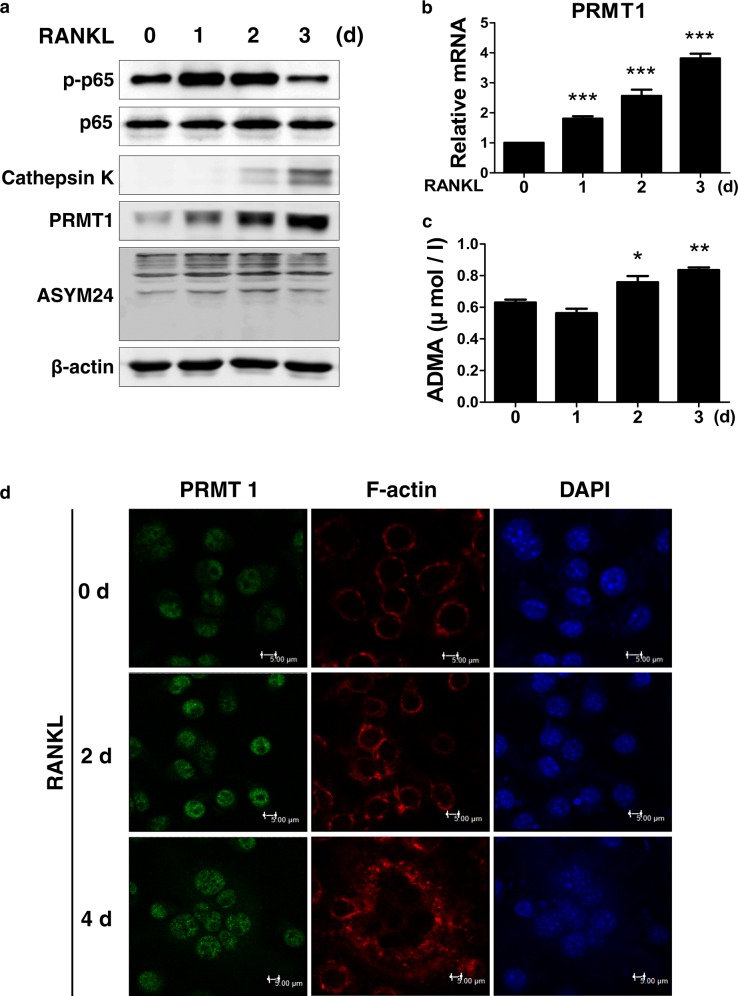
The expression level of PRMT1 increased in RANKL-treated bone marrow-derived macrophages (BMDMs). **a-d** BMDMs were cultured with M-CSF (30 ng/ml) and RANKL (100 ng/ml) for the indicated time periods to induce differentiation into mature osteoclasts. **a** Cellular proteins were extracted and subjected to western blotting with the indicated antibodies. **b** Results of real-time PCR showing the mRNA expression level of PRMT1. The results were normalized to the expression level of β-actin. **c** Expression level of ADMA in a culture supernatant determined by ELISA. The results are expressed as the mean ± SD. *p < 0.05, **p < 0.01, ***p < 0.001 vs. 0 d. **d** BMDMs were labeled with anti-PRMT1 and a fluorescein isothiocyanate (FITC)-conjugated secondary antibody. F-actin ring formation was stained with Alexa Fluor 594-phalloidin and detected using a confocal microscope. The representative images are from at least three independent experiments. Scale bar: 5 μm

To examine the pattern of expression of PRMT1 during osteoclastogenesis, BMDMs were treated with M-CSF and RANKL for the indicated periods of time. BMDMs differentiated into mature osteoclasts after culturing with M-CSF and RANKL for 3 days. As shown in Fig. 1a, RANKL treatment increased the expression of p-p65 at the early stage and of cathepsin K protein at the late stage of osteoclast differentiation, which was consistent with previous studies^25,26^. Remarkably, the protein levels of PRMT1 and ADMA increased in response to treatment with RANKL during osteoclast differentiation (Fig. 1a). The PRMT1 mRNA expression level and ADMA concentration in the culture supernatant also increased in the differentiated cells treated with RANKL (Figs. 1b, c). As shown in Fig. 1d, M-CSF and RANKL treatment induced a well-defined F-actin ring formation, which is associated with the attachment of osteoclasts to the bone surface and this is concomitant with an increase in PRMT1 expression in the nucleus.

### PRMT1 is critical for RANKL-induced osteoclast differentiation

**Fig. 2:**
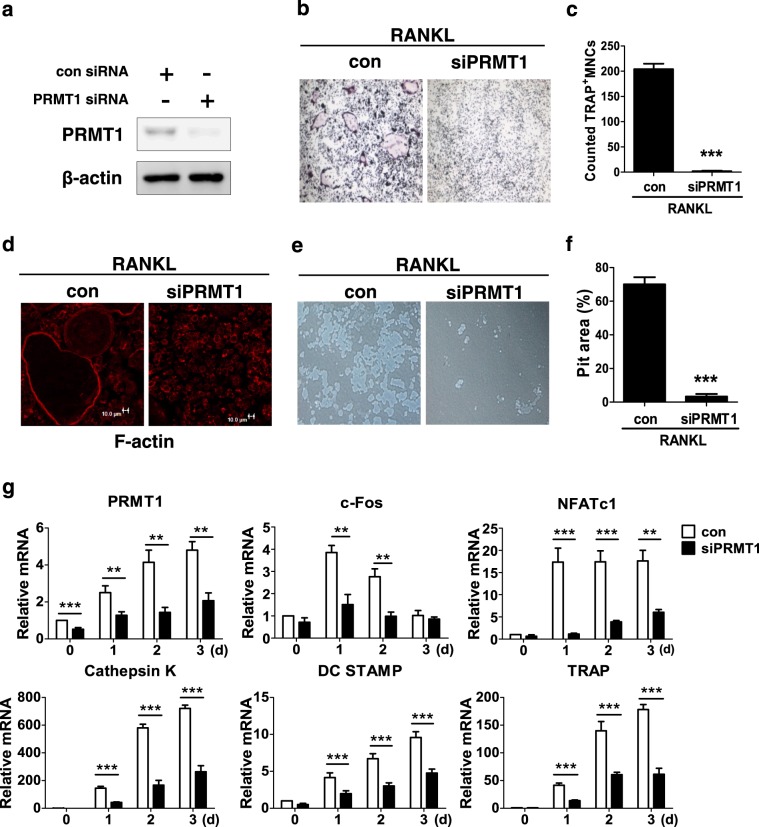
PRMT1 knockdown attenuates RANKL-induced osteoclast function. **a-g** BMDMs were transfected with control siRNA (con) or PRMT1 siRNA (siPRMT1) according to the forward transfection method for 2 days, and these transfected cells were incubated with RANKL (100 ng/ml) in the presence of M-CSF (30 ng/ml) for an additional 3 days. **a** The cell extracts were subjected to a western blot analysis with PRMT1 and β-actin antibodies. **b**, **c** The cells were stained for TRAP activity, and the TRAP-positive multinucleated cells were counted to determine osteoclast numbers. **d** The cells were fixed and stained with Alexa Fluor 594-phalloidin for F-actin. **e**, **f** The cells were cultured on an Osteo Assay Surface Plate for 3 days and then removed. Resorption pits were visualized under light microscopy, and the percentage was obtained using the image J program. Representative images are from at least three independent experiments. **g** Total RNA was extracted from the cells, and the levels of the indicated mRNAs were measured using real-time PCR. The results are normalized to the β-actin level. The results are expressed as the mean ± SD. **p < 0.01, ***p < 0.001

To investigate the role of PRMT1 in RANKL-induced osteoclastogenesis, BMDMs were transfected with con or PRMT1 siRNA and were cultured for 3 days in the presence of M-CSF and RANKL. Control siRNA did not affect PRMT1 expression, whereas PRMT1 siRNA significantly reduced its expression, demonstrating the effectiveness of PRMT1 siRNA (Fig. 2a). Remarkably, there was a significant reduction in the number of TRAP-positive MNCs that differentiated from PRMT1-silenced BMDMs (Figs. 2b, c). Moreover, siRNA specific for PRMT1 inhibited F-actin formation and bone resorption (Figs. 2d, e, f). The effect of PRMT1 on the gene expression of osteoclastic markers such as *c-fos, NFATc1, Cathepsin K*, *DC STAMP* and *TRAP* was further investigated in RANKL-stimulated BMDMs. In a manner consistent with the results of the assays for TRAP activity and bone resorption, RANKL-induced mRNA expression levels were downregulated in PRMT1 siRNA-transfected cells compared with cells with control siRNA (Fig. 2g), emphasizing the positive role of PRMT1 in RANKL-induced osteoclast differentiation.

### Haploinsufficiency of PRMT1 reduces RANKL-induced osteoclastogenesis

**Fig. 3:**
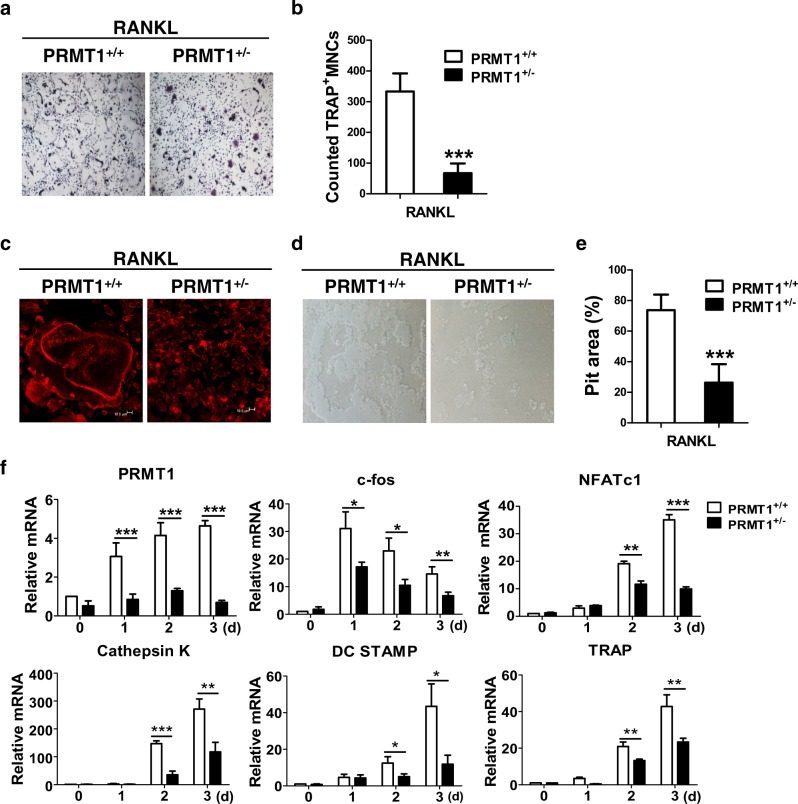
RANKL-induced osteoclastic differentiation and bone resorption alleviated in BMDMs from PRMT1^-^haploinsufficient mice. **a-f** BMDMs from *PRMT1*^*+/+*^ and *PRMT1*^+/-^ mice were incubated with M-CSF (30 ng/ml) and RANKL (100 ng/ml). **a**, **b** The cells were stained for TRAP activity, and the number of osteoclasts was counted after staining for TRAP activity. **c** The cells were fixed and stained with Alexa Fluor 594-phalloidin for F-actin. **d**, **e** BMDMs from *PRMT1*^*+/+*^ and *PRMT1*^+/-^ mice were cultured on an Osteo Assay Surface Plate for 3 days and then removed. Resorption pits were visualized with light microscopy, and the percentage was obtained using the image J program. The representative images are from at least three independent experiments. **f** The levels of the indicated mRNAs were measured using real-time PCR analysis and were then normalized to the β-actin level. The results are expressed as the mean ± SD. *p < 0.05, **p *<* 0.01, ***p < 0.001

To confirm the involvement of PRMT1 in the process of osteoclast differentiation, BMDMs derived from *PRMT1*^+/+^ and *PRMT1*^+/-^ mice were differentiated with M-CSF and RANKL and subjected to TRAP staining or were cultured on an Osteo Assay surface plate. Consistent with results from other experiments using siRNA, the number of TRAP-positive cells was lower in *PRMT1*^*+/-*^ cultures than in *PRMT1*^*+/+*^ cultures (Figs. 3a, b). Moreover, the F-actin ring staining results showed that actin ring formation was blocked in *PRMT1*^*+/-*^ cultures (Fig. 3c). Furthermore, large bone resorption pits were observed in *PRMT1*^*+/+*^ cultures, whereas the resorption areas decreased in *PRMT1*^*+/-*^ cultures (Figs. 3d, e), suggesting that the lack of PRMT1 delayed fusion in mature osteoclasts. Compared with those in *PRMT1*^*+/+*^ cultures, the expression of osteoclast differentiation-associated genes such as *c-fos*, *NFATc1*, *Cathepsin K*, *DC STAMP* and *TRAP* were lower in *PRMT1*^*+/-*^ cultures (Fig. 3f).

### JNK MAPK is essential to increase the expression of PRMT1 during RANKL-induced osteoclast differentiation

**Fig. 4:**
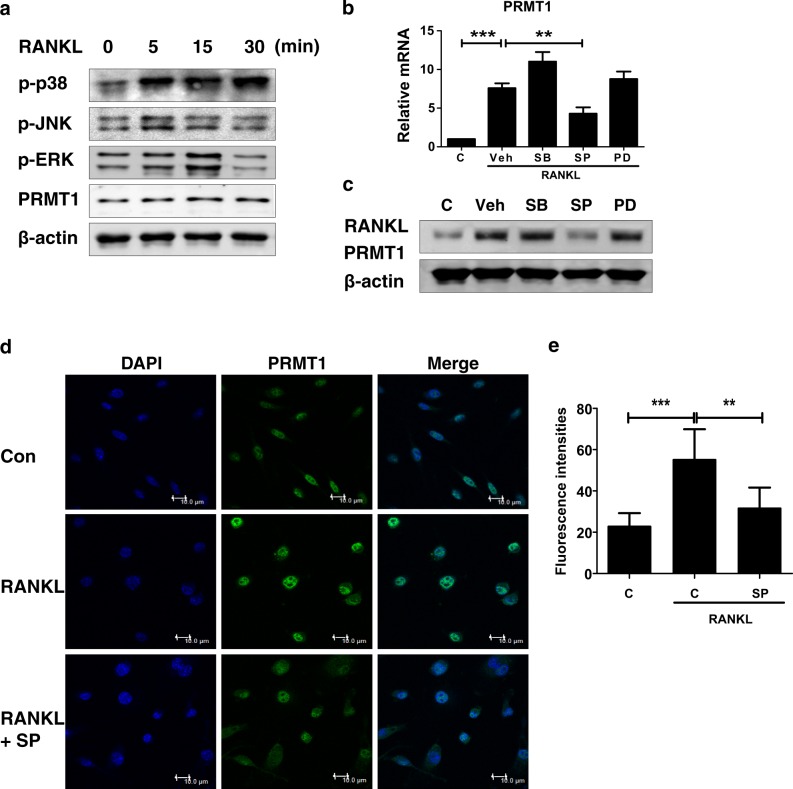
RANKL treatment increases PRMT1 expression via JNK-dependent signaling in osteoclast differentiation. **a** BMDMs were stimulated by RANKL (100 ng/ml) at the indicated time points and were subjected to western blotting with the indicated antibodies. **b-d** The BMDMs were pre-treated with DMSO (Veh), SB 203580 (SB, 10 μM), SP 600125 (SP, 10 μM) or PD 98059 (PD, 10 μM) in the presence of M-CSF (30 ng/ml). After 30 min, the cells were stimulated with RANKL (100 ng/ml) for 24 h. **b** PRMT1 protein expression was examined via western blotting and **c** its mRNA level was measured with a real-time PCR analysis normalized to the β-actin level. The results are expressed as the mean ± SD. **p *<* 0.01, ***p < 0.001. **d** The cells were labeled with anti-PRMT1 and fluorescein isothiocyanate (FITC)-conjugated secondary antibody, and the nuclei were stained with DAPI and observed under a confocal microscope. Representative images are from at least three independent experiments. Scale bar: 10 μm. **e** Fluorescence intensities were obtained from the mean value of the selected regions in the image using Leica Application Suite Advanced Fluorescence (LAS AF) software (Leica Microsystems). The results are expressed as the mean ± SD. **p < 0.01, ***p < 0.001

We further sought to determine the molecular mechanism regulating PRMT1 expression during osteoclastogenesis. MAPKs have been shown to be activated during the early stages of RANKL-induced osteoclast differentiation and are responsible for activating the related transcriptional factors and the expression of relevant genes^27^. Consistently, all three MAPKs, including p38, JNK and ERK, were activated within 15 min after RANKL treatment in BMDMs, whereas PRMT1 expression was not influenced (Fig. 4a). To elucidate the relationship between MAPK and PRMT1 in RANKL-treated BMDMs, BMDMs were treated with SB 203580 (a p38 inhibitor), SP 600125 (a JNK inhibitor) or PD 98059 (an ERK inhibitor) in the presence of M-CSF and RANKL for 24 h. An inhibitor assay revealed that JNK, but not p38 or ERK, was essential for gene and protein expression of PRMT1 in RANKL-treated BMDMs (Figs. 4b, c). Moreover, treatment with SP 600125, a specific inhibitor of JNK, inhibited the nuclear expression of PRMT1 in RANKL-treated BMDMs (Figs. 4d, e). These results suggest that RANKL induces PRMT1 expression in osteoclast differentiation via JNK signaling pathways.

### PRMT1 interacts with the NF-κB subunit p65 during RANKL-mediated osteoclastogenesis

**Fig. 5:**
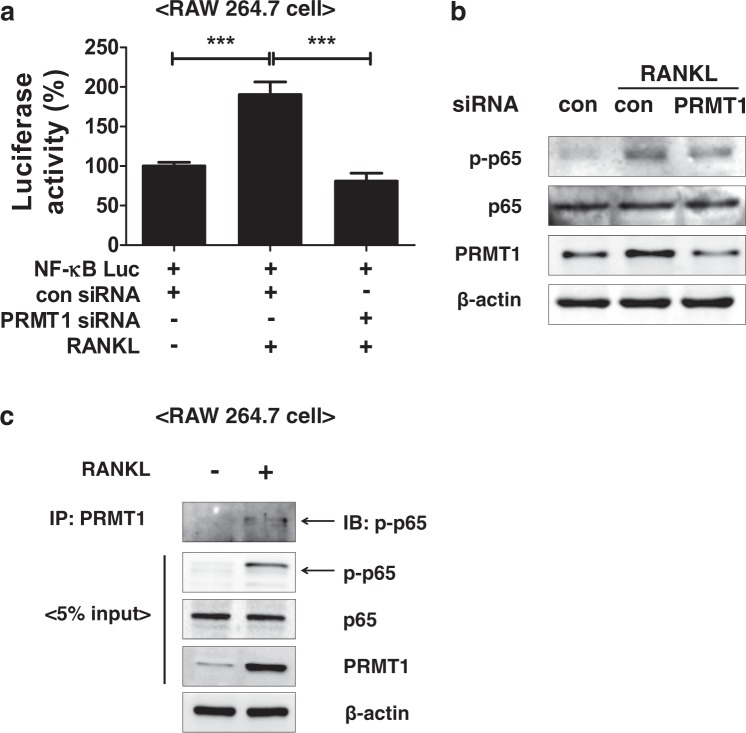
PRMT1 interacts with NF-κB subunit p65 during RANKL-mediated osteoclastogenesis. **a** RAW 264.7 cells were transfected with control or PRMT1 siRNA according to the forward transfection method. After 24 h, the cells were transfected with pGL4.32-*luc2P*/NF-κB-RE and β-galactosidase plasmids. After 24 h of transfection, the cells were treated with RANKL (100 ng/ml) in the presence of M-CSF (30 ng/ml) for 24 h and then lysed with passive lysis buffer for the luciferase assay. Luciferase activity was measured and normalized to β-galactosidase activity. The data are presented as the mean ± SD. ***p < 0.001. **b** BMDMs were transfected with control or PRMT1 siRNA and treated with RANKL for 24 h. Levels of phosphor- and regular p65, PRMT1, and actin were examined by western blot analysis. **c** RAW 264.7 cells were incubated with or without RANKL (100 ng/ml) in the presence of M-CSF (30 ng/ml) for 24 h and immunoprecipitated with anti-PRMT1

Hassa et al. reported that PRMT1 can directly bind to the NF-κB subunit p65 and promote its activation^28^. To examine the role of PRMT1 in RANKL-induced NF-κB activation, a luciferase reporter assay was performed in RAW 264.7 cells, a macrophage cell line with the capacity to form osteoclast-like cells. As shown in Fig. 5a, the transcriptional activity of NF-κB was obviously increased in the cells after treatment with RANKL. However, the increase in NF-κB activity was inhibited by PRMT1 siRNA (Fig. 5a). Western blot analysis also revealed that transient silencing of PRMT1 suppressed the phosphorylation of p65 in response to RANKL in BMDMs (Fig. 5b). We next determined whether PRMT1 directly interacts with the NF-κB subunit p65 in RANKL-treated cells using an immunoprecipitation experiment. The results showed that RANKL treatment leads to an interaction between PRMT1 and a p65 subunit (Fig. 5c). As NF-κB regulates NFATc1 activation and osteoclastogenesis-related gene expression^29^, it is likely that PRMT1 contributes to osteoclast differentiation by regulating NF-κB activity.

### PRMT1 is essential for osteoclastic activity and bone loss in OVX mice

**Fig. 6:**
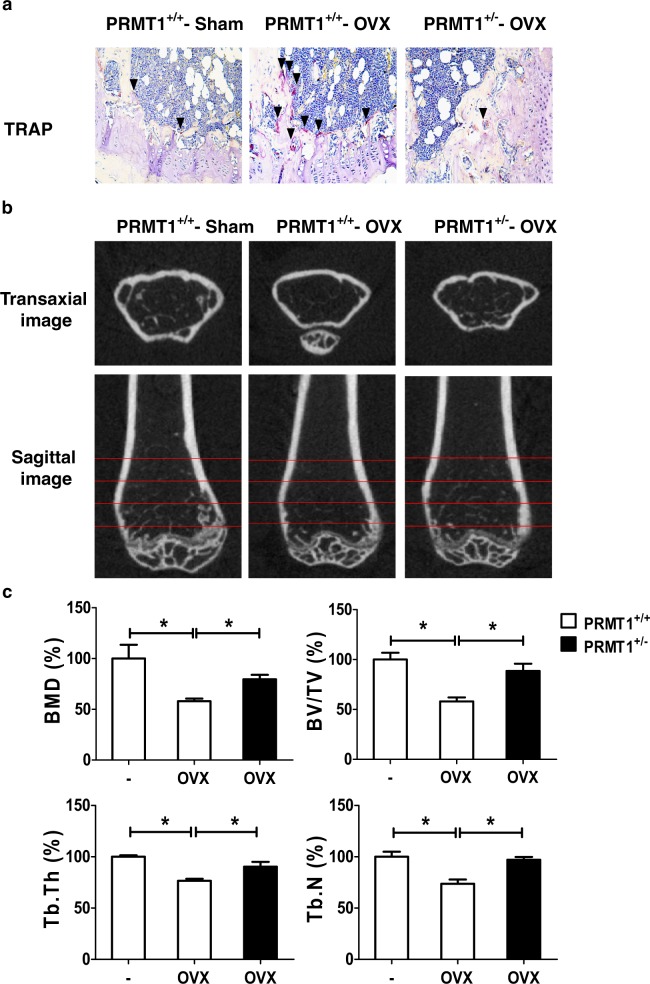
PRMT1 haploinsufficiency mice exhibit an anti-osteoporotic bone phenotype. **a** TRAP staining of distal portions of the femora were performed using leukocyte acid phosphatase kits and observed under a microscope (arrow). **b** The representative two-dimensional images of the femurs are shown. **c** The bone volume per tissue volume (BV/TV), trabecular number (Tb. N), trabecular thickness (Tb. Th) and bone mineral density (BMD) were assessed from μCT measurements (*n* = 6). *p < 0.05

To evaluate the in vivo effect of PRMT1 on the phenotype of osteoporosis and osteoclastic activity, OVX or sham surgery was performed for WT and *PRMT1*^*+/-*^ mice. Eight weeks after OVX, histomorphometric and μCT analyses were performed on the trabecular bone of the distal femur. The sagittal histomorphology paraffin section of the femur was subjected to TRAP staining. Osteoclastic activity was remarkably enhanced in the metaphysis of WT OVX mice but not in *PRMT1*^*+/-*^ OVX mice (Fig. 6a). A micro-CT morphometric analysis revealed that OVX led to a noticeable reduction in the BMD, BV/TV, Tb. N and Tb. Th in WT mice (Figs. 6b, c). However, such reductions were abrogated in *PRMT1*^+/-^ OVX mice (Figs. 6b, c). These results indicate that the deficiency of PRMT1 may protect mice from OVX-induced bone loss.

### Estrogen downregulates RANKL-induced PRMT1 expression

**Fig. 7:**
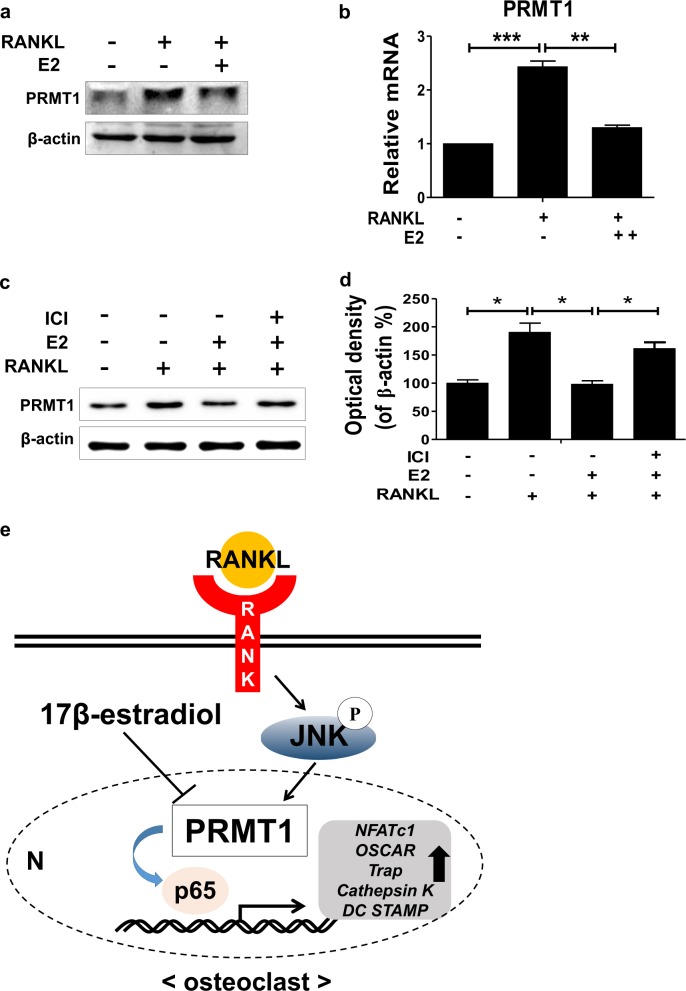
17β-Estrogen decreases RANKL-induced PRMT1 expression in BMDMs. **a**, **b** BMDMs were pre-treated with or without E2 (1 μM) for 2 h and then treated with RANKL (100 ng/ml) for 24 h. **a** PRMT1 protein expression was examined via western blotting. **b** The level of PRMT1 mRNA was measured via real-time PCR and was then normalized to the β-actin level. **c**, **d** BMDMs were pre-treated with E2 (1 μM) with or without pre-treatment by ICI 182,780 (10 μM). After 2 h, the cells were treated with RANKL (100 ng/ml) for 24 h. The proteins were extracted and subjected to western blot analysis with PRMT1 and β-actin antibodies. The data are presented as the mean ± SD. *p < 0.05, **p *<* 0.01, ***p < 0.001. **e** Schematic showing the mechanism of PRMT1 during osteoclastogenesis

Given that *PRMT1*^+/-^ OVX mice repress osteoclast activity and bone loss and that stimulation with E2 is sufficient to block the differentiation of osteoclasts by RANKL^30^, we further investigated whether PRMT1 expression might be influenced by 17β-estrogen (E2) during osteoclast differentiation in vitro. BMDMs were pre-treated with E2 and were subsequently treated with RANKL and PRMT1 protein, and the PRMT1 mRNA levels were determined. The results showed that RANKL-mediated the expression of the gene, and the protein expression level of PRMT1 was suppressed by E2 treatment in BMDMs (Figs. 7a, b). Moreover, ICI 182,780, an ER antagonist, abolished the inhibitory effect of E2 on PRMT1 expression (Figs. 7c, d). These findings suggest that E2 may exert a protective effect against osteoporosis by downregulating PRMT1 expression.

## Discussion

In the present study, we have demonstrated for the first time that PRMT1 is involved in RANKL-induced osteoclastogenesis and in OVX-induced bone loss using PRMT1 haploinsufficent mice. PRMT1 expression increased significantly during RANKL-induced osteoclast differentiation, and it served as an intermediate molecule in the differentiation of the osteoclasts. In addition, OVX-induced osteoporosis improved in PRMT1 haploinsufficient (*PRMT1*^*+/-*^) mice, and these results suggest that PRMT1 may contribute to osteoclast differentiation and osteoporosis.

Previous studies have provided evidence that ADMA, the product of type 1 PRMTs, might be implicated in the onset of osteoporosis. Kanazawa et al. reported that in patients with diabetes, the level of serum dimethylarginine is associated with the presence of vertebral fractures^31^. Lu et al. demonstrated that an increase in serum ADMA levels is associated with an age-related decrease in the BMD of rats^32^. In the present study, we revealed that the ADMA levels increased in cell culture media during RANKL-induced osteoclast differentiation. Although several studies have reported that ADMA is associated with various metabolic diseases^33,34^, further studies are needed to reveal the precise role of ADMA as a risk factor in osteoclast differentiation and osteoporosis. In addition, they should clarify the clinical relevance of ADMA levels in osteoporotic patients.

Despite having a major role in the arginine methylation of nuclear proteins, very little is known about the significance of the intracellular localization of PRMT1. Herrmann et al. reported that PRMT1 is observed more predominantly in the cytoplasm than in the nucleus under normal cell (HEK293) conditions^35^. However, this group has recently hypothesized that the subcellular distribution of PRMT1 could be cell-type specific. They have shown that PRMT1 is predominantly cytoplasmic in U2O2 osteosarcoma cells, whereas it is enriched in the nucleus of MCF7 breast cancer and HeLa cervix carcinoma cell lines^36^. They suggested that these differences in the localization of PRMT1 are due to the variability in cell lines related to the expression of substrate proteins that need to be methylated. Park et al. reported that palmitate treatment increases the amount of PRMT1 primarily in the nucleus of glomerular mesangial cells, which is expected to increase the transcription levels of the target genes involved in apoptosis and ER stress^37^. Consistent with their results, our results showed that RANKL increased PRMT1 expression in the nucleus of BMDMs. The major targets of PRMT1 have been found to be nuclear proteins that can enhance the transcriptional activation in a manner that requires PRMT1 activity^38^. This suggests that PRMT1 might act as a transcriptional cofactor or methylate histone 4 on arginine 3 to increase the transcription levels of the target genes, thus inducing osteoclast differentiation. In this regard, Hassa et al. reported that PRMT1 can synergistically co-activate NF-κB-dependent gene expression via the human immunodeficiency virus-1 LTR promoter and interact directly with the NF-κB subunit p65 in the nucleus^28^. The activation of NF-κB is essential for RANKL-induced osteoclast signaling, which regulates osteoclast formation, function and survival^39^. RANKL-induced NF-κB activation results in the degradation of inhibitor of kappa B alpha (IκBα), and such degradation allows the NF-κB subunit p65 to enter the nucleus and bind to DNA target sites^40^. NF-κB binds to NFATc1 promoter regions and initiates the expression of osteoclast-related genes including cathepsin K, DC STAMP, TRAP and NFATc1 itself^41,42^. Taken together, our results indicate that RANKL-induced PRMT1 may regulate osteoclast differentiation by regulating NF-κB activity in the nucleus by binding to subunit p65. This finding is supported by the fact that NF-κB (p65) activity was diminished by PRMT1 siRNA in RANKL-treated BMDMs.

RANKL treatment leads to early activation of MAPKs in BMDMs, which is critical for osteoclastogenesis including cell proliferation, differentiation and osteoclast-related gene expression^41,43,44–46^. Consistently, in the present study, an increased phosphorylation of p38, JNK and ERK was detected in RANKL-treated BMDMs within 15 min, but there was no remarkable change in the protein level of PRMT1. Interestingly, the inhibition of JNK, but not p38 and ERK, suppressed RANKL-induced PRMT1 expression, and this was supported by a previous study showing that PRMT1 expression was increased in human lung epithelial cells undergoing hypoxia-induced apoptosis in a JNK-dependent manner^47^. In addition, RANKL binding to RANK triggers JNK activation, which subsequently increases the transcription of the *c-fos* gene during the early stage of osteoclast differentiation^48^. *c-fos*-deficient mice have been reported to develop osteopetrosis^49^, and *c-fos* regulates various osteoclast-specific genes through cooperation with NFATc1^42^. In the present study, we showed that RANKL-induced *c-fos* expression diminished in BMDMs transfected with PRMT1 siRNA and in those derived from *PRMT1*^+/-^ mice. It is likely that JNK-mediated signaling is responsible for an increase in the expression of PRMT1, which regulates the expression of genes related to osteoclast differentiation, such as *c-fos*.

Postmenopausal osteoporosis is one of the most common metabolic bone diseases associated with estrogen deficiency. Bone loss caused by estrogen deficiency is primarily due to an increase in the osteoclastic bone resorption in both humans and experimental animals^50^. In the present study, we investigated the role of PRMT1 in osteoporosis in vivo by comparing the osteoclastic activity and BMD between WT and *PRMT1*^*+/-*^ mice with OVX, given that PRMT1 homozygous knockout mice are embryonically lethal^51^. The in vivo results showed that the osteoclastic activity was lower and BMD was improved in *PRMT1*^*+/-*^ mice, indicating that PRMT1 plays an important role in bone loss induced by estrogen deficiency in vivo. In fact, RANKL-induced PRMT1 expression in BMDMs was inhibited after E2 treatment, which is known to exert an inhibitory effect on RANKL-induced osteoclast differentiation by downregulating the activation of JNK^52^. In accordance with this, Schulze et al. suggested that ADMA levels were increased in women after menopause in comparison with men and that ADMA is decreased during normal pregnancy, which is a hyperestrogenic state^53,54^. These data imply that estrogen may regulate the ADMA level in physiological states by regulating PRMT1 expression because PRMT1 is known to largely contribute to ADMA formation. Collectively, these results support the idea that estrogen suppresses osteoclast differentiation through downregulation of RANKL-induced JNK activation and PRMT1 expression in normal physiology.

In conclusion, our results showed that PRMT1 expression was enhanced in the nucleus of RANKL-treated BMDMs via a JNK-mediated signaling pathway (Fig. 7e). PRMT1 was essential for RANKL-induced osteoclastic activity, bone resorption and upregulation of osteoclastogenesis-related genes. In addition, the deficiency of PRMT1 reduced the in vivo osteoclastic activity and bone loss in OVX mice. Thus, PRMT1 can be a therapeutic target to treat the development and progression of osteoporosis.

## References

[1] Karsenty G (2003). The complexities of skeletal biology. Nature.

[2] Li X (2016). Disturbed MEK/ERK signaling increases osteoclast activity via the Hedgehog-Gli pathway in postmenopausal osteoporosis. Pro. Biophys. Mol. Biol..

[3] McNamara LM (2010). Perspective on post-menopausal osteoporosis: establishing an interdisciplinary understanding of the sequence of events from the molecular level to whole bone fractures. J. R. Soc. Interface.

[4] Melton LJ, Chrischilles EA, Cooper C, Lane AW, Riggs BL (2005). How many women have osteoporosis?. J. Bone Miner. Res..

[5] Anderson GL (2004). Effects of conjugated equine estrogen in postmenopausal women with hysterectomy: the Women’s Health Initiative randomized controlled trial. J. Am. Med Assoc..

[6] Pixley FJ, Stanley ER (2004). CSF-1 regulation of the wandering macrophage: complexity in action. Trends Cell Biol..

[7] Kim H (2009). Selective inhibition of RANK blocks osteoclast maturation and function and prevents bone loss in mice. J. Clin. Invest..

[8] Huang H (2006). Osteoclast differentiation requires TAK1 and MKK6 for NFATc1 induction and NF-kappaB transactivation by RANKL. Cell Death Differ..

[9] Matsuo K (2004). Nuclear factor of activated T-cells (NFAT) rescues osteoclastogenesis in precursors lacking c-Fos. J. Biol. Chem..

[10] Yang SH, Sharrocks AD, Whitmarsh AJ (2013). MAP kinase signalling cascades and transcriptional regulation. Gene.

[11] Chen Y (2009). PRMT-1 and DDAHs-induced ADMA upregulation is involved in ROS- and RAS-mediated diabetic retinopathy. Exp. Eye Res..

[12] Iberg AN (2008). Arginine methylation of the histone H3 tail impedes effector binding. J. Biol. Chem..

[13] Yang Y, Bedford MT (2013). Protein arginine methyltransferases and cancer. Nat. Rev. Cancer.

[14] Tang J, Kao PN, Herschman HR (2000). Protein-arginine methyltransferase I, the predominant protein-arginine methyltransferase in cells, interacts with and is regulated by interleukin enhancer-binding factor 3. J. Biol. Chem..

[15] Kim DI (2015). PRMT1 and PRMT4 regulate oxidative stress-induced retinal pigment epithelial cell damage in SIRT1-dependent and SIRT1-independent manners. Oxid. Med. Cell Longev..

[16] Mathioudaki K (2008). The PRMT1 gene expression pattern in colon cancer. Br. J. Cancer.

[17] Park MJ (2014). Thioredoxin-interacting protein mediates hepatic lipogenesis and inflammation via PRMT1 and PGC-1alpha regulation in vitro and in vivo. J. Hepatol..

[18] Siroen MP (2006). The clinical significance of asymmetric dimethylarginine. Annu. Rev. Nutr..

[19] Sydow K, Mondon CE, Cooke JP (2005). Insulin resistance: potential role of the endogenous nitric oxide synthase inhibitor ADMA. Vasc. Med..

[20] Lu R, Hu CP, Wu XP, Liao EY, Li YJ (2002). Effect of age on bone mineral density and the serum concentration of endogenous nitric oxide synthase inhibitors in rats. Comp. Med..

[21] Holden DP, Cartwright JE, Nussey SS, Whitley GS (2003). Estrogen stimulates dimethylarginine dimethylaminohydrolase activity and the metabolism of asymmetric dimethylarginine. Circulation.

[22] Kemeny-Beke A (2011). Increased production of asymmetric dimethylarginine (ADMA) in ankylosing spondylitis: association with other clinical and laboratory parameters. Jt. Bone Spine.

[23] Morales Y, Caceres T, May K, Hevel JM (2016). Biochemistry and regulation of the protein arginine methyltransferases (PRMTs). Arch. Biochem. Biophys..

[24] Dong Y (2017). Inhibition of PRMT5 suppresses osteoclast differentiation and partially protects against ovariectomy-induced bone loss through downregulation of CXCL10 and RSAD2. Cell Signal..

[25] Stoch SA, Wagner JA (2008). Cathepsin K inhibitors: a novel target for osteoporosis therapy. Clin. Pharmacol. Ther..

[26] Vaira S (2008). RelA/p65 promotes osteoclast differentiation by blocking a RANKL-induced apoptotic JNK pathway in mice. J. Clin. Invest..

[27] Wang X (2015). Amiloride inhibits osteoclastogenesis by suppressing nuclear factor-kappaB and mitogen-activated protein kinase activity in receptor activator of nuclear factor-kappaB-induced RAW 264.7 cells. Mol. Med. Rep..

[28] Hassa PO, Covic M, Bedford MT, Hottiger MO (2008). Protein arginine methyltransferase 1 coactivates NF-kappaB-dependent gene expression synergistically with CARM1 and PARP1. J. Mol. Biol..

[29] Boyce BF, Xiu Y, Li J, Xing L, Yao Z (2015). NF-kappaB-mediated regulation of osteoclastogenesis. Endocrinol. Metab. (Seoul).

[30] Robinson LJ (2009). Estrogen inhibits RANKL-stimulated osteoclastic differentiation of human monocytes through estrogen and RANKL-regulated interaction of estrogen receptor-alpha with BCAR1 and Traf6. Exp. Cell Res..

[31] Kanazawa I (2010). Relationships between dimethylarginine and the presence of vertebral fractures in type 2 diabetes mellitus. Clin. Endocrinol. (Oxf.).

[32] Franzoso G (1997). Requirement for NF-kappaB in osteoclast and B-cell development. Genes Dev..

[33] Hanai K (2009). Asymmetric dimethylarginine is closely associated with the development and progression of nephropathy in patients with type 2 diabetes. Nephrol. Dial. Transplant..

[34] Park MJ, Oh KS, Nho JH, Kim GY, Kim DI (2016). Asymmetric dimethylarginine (ADMA) treatment induces apoptosis in cultured rat mesangial cells via endoplasmic reticulum stress activation. Cell Biol. Int..

[35] Herrmann F, Lee J, Bedford MT, Fackelmayer FO (2005). Dynamics of human protein arginine methyltransferase 1(PRMT1) in vivo. J. Biol. Chem..

[36] Herrmann F, Pably P, Eckerich C, Bedford MT, Fackelmayer FO (2009). Human protein arginine methyltransferases in vivo--distinct properties of eight canonical members of the PRMT family. J. Cell Sci..

[37] Park MJ, Han HJ, Kim DI (2017). Lipotoxicity-induced PRMT1 exacerbates mesangial cell apoptosis via endoplasmic reticulum stress. Int J. Mol. Sci..

[38] Wang H (2001). Methylation of histone H4 at arginine 3 facilitating transcriptional activation by nuclear hormone receptor. Science.

[39] Soysa NS, Alles N (2009). NF-kappaB functions in osteoclasts. Biochem. Biophy. Res. Commun..

[40] Asagiri M (2005). Autoamplification of NFATc1 expression determines its essential role in bone homeostasis. J. Exp. Med..

[41] Boyle WJ, Simonet WS, Lacey DL (2003). Osteoclast differentiation and activation. Nature.

[42] Takayanagi H (2002). Induction and activation of the transcription factor NFATc1 (NFAT2) integrate RANKL signaling in terminal differentiation of osteoclasts. Dev. Cell.

[43] Ikeda F (2004). Critical roles of c-Jun signaling in regulation of NFAT family and RANKL-regulated osteoclast differentiation. J. Clin. Invest..

[44] Lee ZH, Kim HH (2003). Signal transduction by receptor activator of nuclear factor kappa B in osteoclasts. Biochem. Biophys. Res. Commun..

[45] Matsumoto M, Sudo T, Maruyama M, Osada H, Tsujimoto M (2000). Activation of p38 mitogen-activated protein kinase is crucial in osteoclastogenesis induced by tumor necrosis factor. FEBS Lett..

[46] Miyazaki T (2000). Reciprocal role of ERK and NF-kappaB pathways in survival and activation of osteoclasts. J. Cell Biol..

[47] Lim SK (2013). Activation of PRMT1 and PRMT5 mediates hypoxia- and ischemia-induced apoptosis in human lung epithelial cells and the lung of miniature pigs: the role of p38 and JNK mitogen-activated protein kinases. Biochem. Biophys. Res. Commun..

[48] Minden A, Karin M (1997). Regulation and function of the JNK subgroup of MAP kinases. Biochim. Biophys. Acta.

[49] Grigoriadis AE (1994). c-Fos: a key regulator of osteoclast-macrophage lineage determination and bone remodeling. Science.

[50] Riggs BL (2000). The mechanisms of estrogen regulation of bone resorption. J. Clin. Invest..

[51] Choi D (2012). Protein arginine methyltransferase 1 regulates hepatic glucose production in a FoxO1-dependent manner. Hepatology.

[52] Srivastava S (2001). Estrogen decreases osteoclast formation by down-regulating receptor activator of NF-kappa B ligand (RANKL)-induced JNK activation. J. Biol. Chem..

[53] Pettersson A, Hedner T, Milsom I (1998). Increased circulating concentrations of asymmetric dimethyl arginine (ADMA), an endogenous inhibitor of nitric oxide synthesis, in preeclampsia. Acta Obstet. Gynecol. Scand..

[54] Schulze F (2005). Determination of a reference value for N(G), N(G)-dimethyl-L-arginine in 500 subjects. Eur. J. Clin. Invest..

